# Aerosol emission of adolescents voices during speaking, singing and shouting

**DOI:** 10.1371/journal.pone.0246819

**Published:** 2021-02-10

**Authors:** Dirk Mürbe, Martin Kriegel, Julia Lange, Lukas Schumann, Anne Hartmann, Mario Fleischer

**Affiliations:** 1 Department of Audiology and Phoniatrics, Charité – Universitätsmedizin Berlin, Berlin, Germany; 2 Hermann-Rietschel-Institut, Technische Universität Berlin, Berlin, Germany; University Hospital Eriangen at Friedrich-Alexander-University Erlangen-Numberg, GERMANY

## Abstract

Since the outbreak of the COVID-19 pandemic, singing activities for children and young people have been strictly regulated with far-reaching consequences for music education in schools and ensemble and choir singing in some places. This is also due to the fact, that there has been no reliable data available on aerosol emissions from adolescents speaking, singing, and shouting. By utilizing a laser particle counter in cleanroom conditions we show, that adolescents emit fewer aerosol particles during singing than what has been known so far for adults. In our data, the emission rates ranged from 16 P/s to 267 P/s for speaking, 141 P/s to 1240 P/s for singing, and 683 P/s to 4332 P/s for shouting. The data advocate an adaptation of existing risk management strategies and rules of conduct for groups of singing adolescents, like gatherings in an educational context, e.g. singing lessons or choir rehearsals.

## Introduction

Aerosols are liquid or solid particles, which are transported in the air and not influenced by gravitation usually determined by a size less than 5 μm, that escape from the respiratory system during breathing, speaking and singing. Besides droplets, they are widely accepted carriers for the transmission of SARS-CoV-2 viruses [1]. Due to the principles of voice production and the described accumulation of SARS-CoV-2-infections during choir rehearsals [2], it is assumed that singing is connected with increased aerosol emission rates. Recently, increased aerosol emissions during singing in comparison to speaking have been experimentally confirmed for adult singers [3, 4]. Further, an increased aerosol emission rate is found for raising vocal loudness [5]. This results in limitations and specific risk management strategies especially for choir singing during the COVID-19-pandemic. However, data about aerosol emission during singing for adolescents are still missing. But especially for children and young people the restrictions on ensemble and choir singing have far-reaching consequences in addition to severe cultural and financial losses. Singing together is an obligatory part of school education and an important factor for the socio-emotional development of children and young people. This applies not only to music lessons in school, but also to the extracurricular sector with music schools and children and youth choirs. By now, the hygiene and performance concepts rely on aerosol emission rates during singing as collected from adults. For the first time, this pilot study presents data of aerosol formation when young people sing.

## Materials and methods

Four girls and four boys, all 13 years old (except one girl aged 15 years), recruited in July 2020 by targeted call, participated in the study. They were members of a semiprofessional children’s choir (Staats- und Domchor Berlin, Mädchenchor der Singakademie Berlin) and had perennial choir experience between five and nine years. All of them were before puberty voice changes, which was assessed by experienced choral directors. Apart from adolescent age, gender, perennial choir experience and pre-puberty voice status were no further inclusion criteria for this study.

The combination of adolescence and pre-puberty voice status allowed studying a representive group within boys’ and girls’ choir singers with advanced singing experience and cognitive development.

The study was conducted according to the ethical principles based on the WMA Declaration of Helsinki and was approved by the ethic committee of the Charité–Universitätsmedizin Berlin, Germany. Informed written consent was obtained from all subjects and their parents.

The investigations were carried out in a cleanroom (ISO-2-class) at the Hermann Rietschel-Institute, Technische Universität Berlin, accessible through an airlock and equipped with terminal U15-filters.

**Fig 1:**
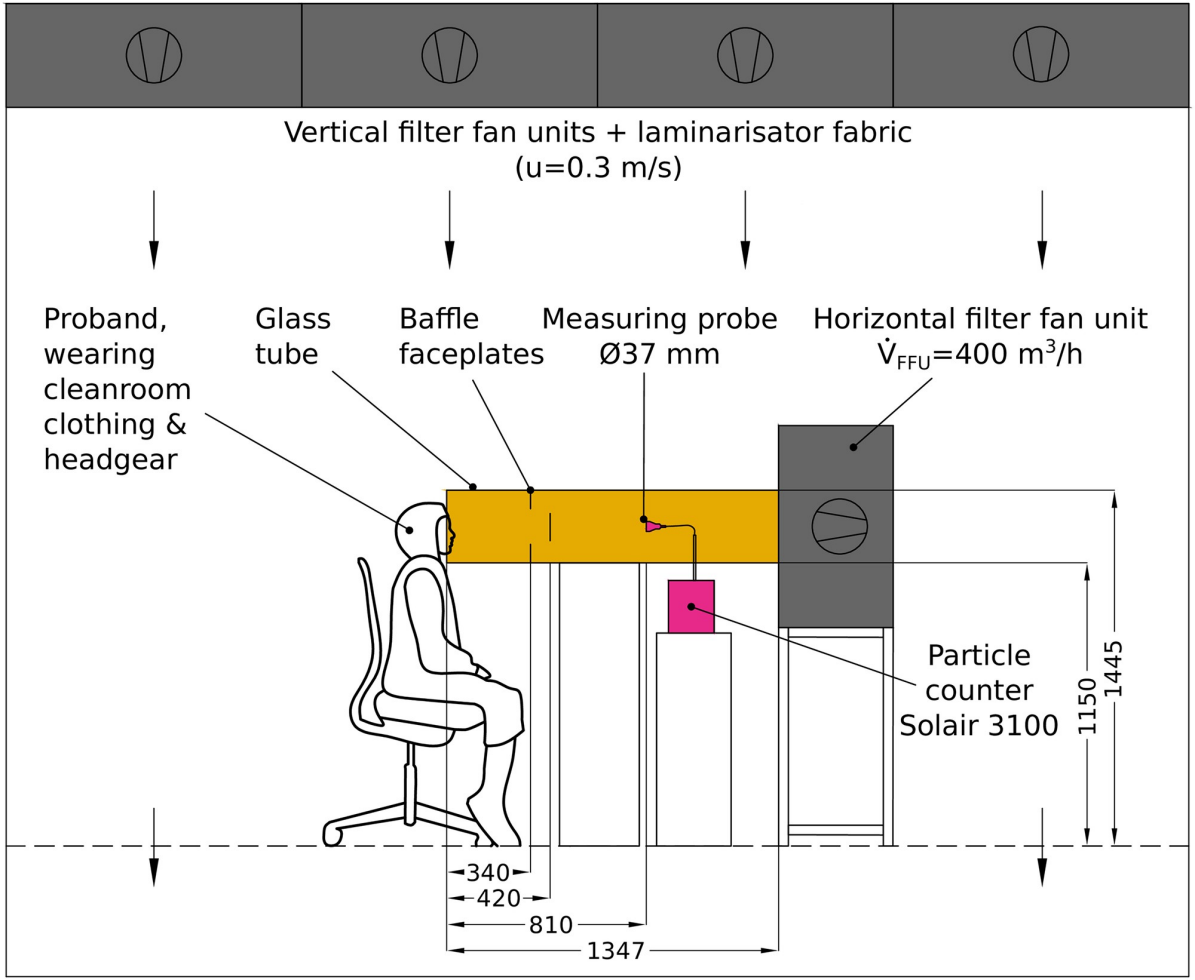
Experimental setup. The subjects sat frontal with their head at the free end of a glass tube (inner diameter 295 mm, length of 1347 mm) during the phonatory tasks. The suction side of a FFU that produced a horizontal volume flow of 400 m^3^/h on average was placed at the opposite end. To achieve a fully developed turbulent flow at the position of the LPC at 810 mm, two baffle plates at distances of 340 mm and 420 mm from the entry were applied. All length units are in mm.

To suppress the thermal plume at the subjects efficiently, the supply air in the whole cleanroom was introduced via a quasi-laminar vertical flow with a velocity of 0.3 m/s (Fig 1) [6]. Further, the room temperature was 295.15±0.5 K, and the relative humidity was 46±2%. The static pressure in the cleanroom was about 20 ⋅ (1±2%) Pa greater than outside the room.

In this highly pure environment, the subjects wore cleanroom clothing and a headgear to further reduce the clothing’s particle emission. To perform the experiments, subjects sat in front of the test equipment, consisting of a glass pipe with a diameter of 295 mm, through which a constant airflow of approximately 400 m^3^/h was generated by suction of a filter fan unit (FFU) (Ziehl-Abegg, Künzelsau, Deutschland) (Fig 1).

The vertical flow in the cleanroom and the glass-pipe construction ensured that emissions from other people (instructor, technical staff), who were necessarily in the cleanroom during measurements, were not directed to a laser particle counter (LPC) (Lighthouse Solair 3100 E, Lighthouse Worldwide Solutions, Fremont, CA).

The sampling probe (37 mm) of the LPC was placed centrally in the pipe. To reach a homogenous particle distribution at the measurement position, two baffles were inserted in the pipe to achieve a sufficient mixing of the particles at the sample point (Fig 1). The position of the LPC within the tube was based on the precedent setup of the measuring system utilizing visual (with stage fog) and quantitative (with an aerosol generator) assessments.

The particle counter was counting with a sampling flow rate of 28.3 l/min with a measuring time increment of 10 seconds. The detected particles were assigned to six size classes (>0.3, >0.5, >1.0, >3.0, >5.0, >10.0 μm) between >0.3 μm—25.0 μm.

According to ISO 21501-4, the counting efficiency for particles of the size 0.3 μm was 50±20%, and for particles of the size 0.5 μm was 100±10%. An initial baseline measurement showed a count rate of <1 particles for 5 minutes. Between the trials and tasks, a time increment of 20-30 s and 60-90 s, respectively, was chosen to avoid remaining particles of the previous task. This was confirmed by the display of a zero count at the LPC.

The emission rate P_M_ was computed based on scaling of the particles measured at the LPC to the volume flow of the whole glass pipe. Apart from the particle measurement, the sound pressure level L_AF_MAX__ was measured via a calibrated sound level meter (CENTER 322_ Datalogger Sound Level Meter, Center Technologies, Houston, TX).

In the first task, the emission rates for three different vocal test conditions were compared: (a) speaking, (b) singing, and (c) shouting. Condition (a) was reading a standardized text (“Der Nordwind und die Sonne” by Äsop), a reference text for voice assessments with balanced phoneme representation. Condition (b) was singing the Swedish folk song “Vem kan segla” in key G-Major, a piece very familiar to the choir singers which could be sung reliably. For condition (c), subjects were asked to cheer enthusiastically about a soccer game goal. The time window for a measured sequence was 30 seconds for test conditions (a) and (b) and 10 seconds for test condition (c). Each test condition was repeated five times.

In the second task, sustained phonation about 10 seconds was performed to investigate the impact of vocal loudness on the emission rate. Subjects were asked to sustain the syllable /*la*/, pitch G4 (392 Hz), at the two loudness conditions soft voice (piano) and loud voice (forte). To facilitate the 10 seconds measurement time, the young people were allowed to take a short breath within the recording and to repeat the syllable.

The emission rates were normalized to the respective time length of the tasks (10 or 30 seconds, respectively) and are time-averaged values.

Statistical analysis, individually handled for the two tasks, was carried out by using linear mixed-effects model (LMEM) analysis in the statistical software R (https://www.r-project.org/) including the package lmerTest [7]. This robust and flexible statistical framework was proven to have a high accuracy for multiple observations for numerous items [8]. For this study, log-valued P_M_ data were incorporated as the response variable and condition as fixed effect. Further, intercepts for subject and by-subject random slopes for the effect of condition were regarded as random effects. P-values were obtained using Satterthwaite’s degree of freedom method. The raw data of this study and the R-script containing statistical analyses are deposited in S1 and S2 Files.

## Results

**Fig 2:**
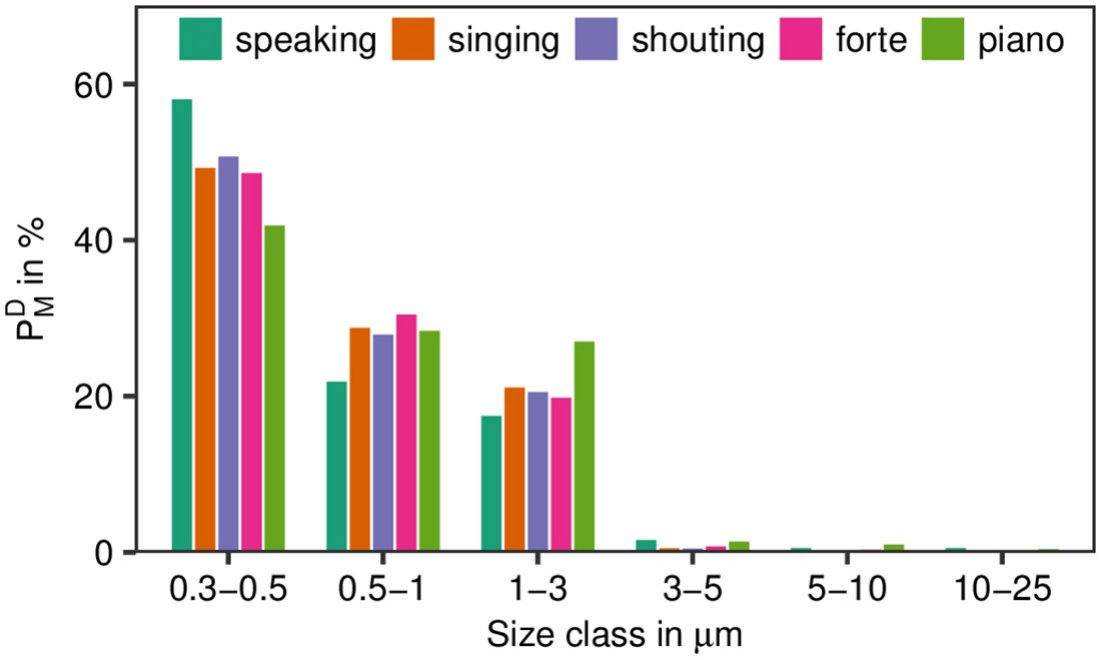
Size distributions. Distributions of emission rates $\text{P}{}_{\text{M}}^{\text{D}}$ for different size classes and test conditions in%. These data were computed by summarizing all P_M_-values for all subjects (separated by test condition) and were normalized to the sum for all size classes dependent on test condition (see S2 File for details).

Within the measuring range between 0.3 μm and 25 μm, about 99% of all measured particles for all test conditions were smaller than 5 μm and more than 70% smaller than 1 μm (Fig 2). With regard to the common understanding to denominate particles with a size smaller than 5 μm as aerosols, the following results are cumulatively given for particles of size 0.3 μm—5 μm.

**Fig 3:**
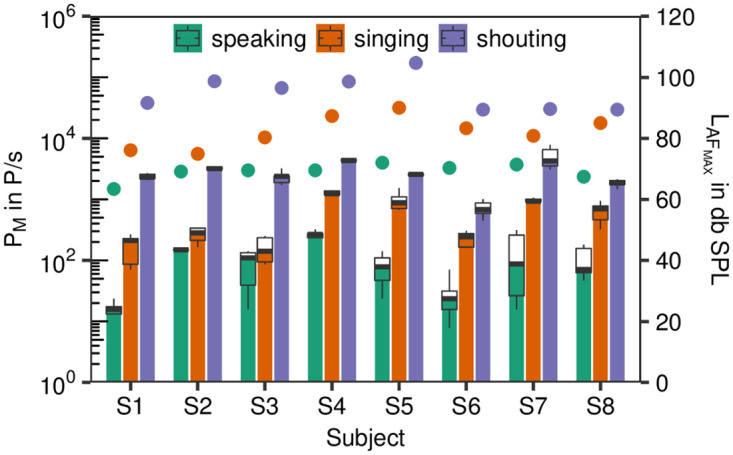
Emission rates. Boxplots of the emission rates (P_M_ in P/s, left y-axis) for the test conditions speaking, singing and shouting for subjects S1-S4 (girls) and S5-S8 (boys). The maximum sound pressure levels (L_AF_MAX__ in db SPL) are also shown (right y-axis) with different colored full circles for the test conditions.

The emission rates P_M_ for speaking were clearly lower than for singing (Fig 3). Whereas the median values for speaking were between 16 P/s (Particles/second) and 267 P/s, this measure was between 141 P/s and 1240 P/s for singing. For shouting, P_M_ was still higher with values from 683 P/s up to 4332 P/s. All subjects showed a clear individual increase in P_M_ for all three conditions.

Linear mixed modeling showed, that these increases in condition were significant (likelihood-ratio test; p<.00001). On average, the ratio of P_M_ between singing and speaking was 5.87±1.28 (standard error). For shouting and speaking, this ratio was 36.22±1.28 (standard error). Both findings were significant (p<.001). Further, P_M_ was positively correlated with the maximum sound pressure level L_AF_MAX__. An increase in one unit in L_AF_MAX__ resulted in an increase in 0.05 units of log_10_(P_M_). This finding was significant (p<.001).

**Fig 4:**
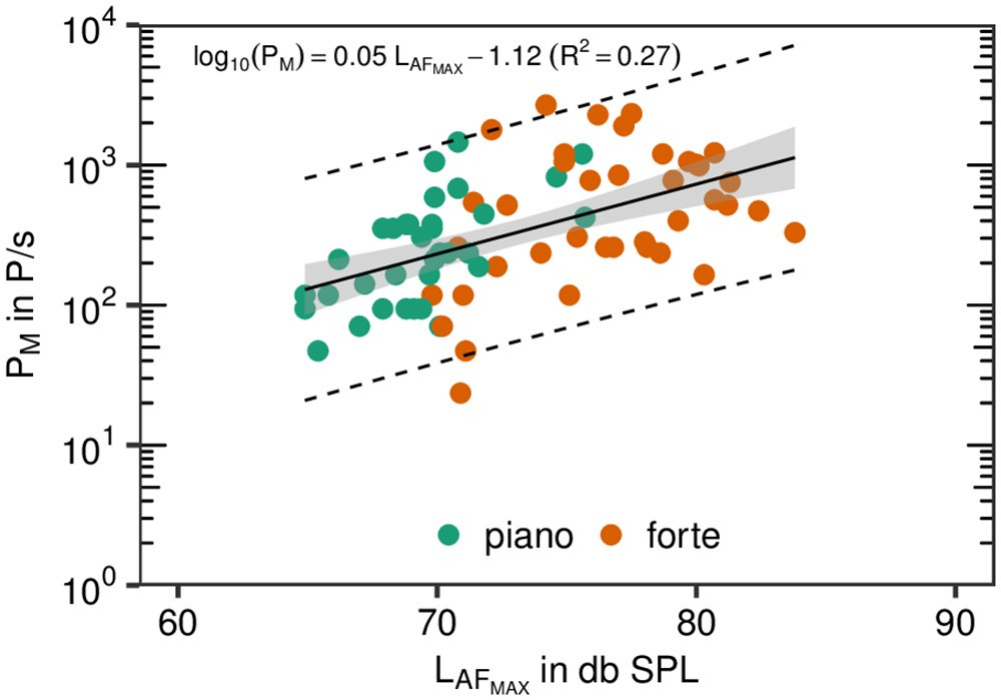
Emission rates vs. sound pressure level. Emission rate P_M_ plotted over maximum sound pressure level L_AF_MAX__ for sustained syllable /*la*/. All five repetitions for the two loudness conditions are represented by colored points as denoted in the legend. The black solid line represents the linear regression (see inset for details), the gray colored area represents the 95% confidence region, whereas the black dashed lines restrict the 95% prediction band.

For the sustained phonation task, the median values for soft phonation (piano) were between 58 P/s and 683 P/s, this measure was between 58 P/s and 1907 P/s for loud phonation (forte). In contrast to the first task, not all subjects showed a clear increase in P_M_ from piano to forte. This finding was mirrored by the results of the linear mixed modeling approach. The increase of P_M_ from piano to forte was 1.91±1.47, whereas the condition was not significant (p = .133). Nevertheless, a positive correlation to L_AF_MAX__ was found (Fig 4), which indicates that the emission rate increases with raising vocal loudness. Similar to the first task, an increase in one unit in L_AF_MAX__ results in an increase in 0.05 units of log_10_(P_M_), which was also significant (p<.001).

## Discussion

The present study confirms higher emission rates of aerosols for singing in comparison to speaking also for adolescents. As for measurements of adult professional singers, a strong intersubject variability of aerosol emission was found for singing adolescents, too. Finally, a positive correlation of particle emission with vocal loudness was confirmed, in particular reflected by the shouting condition. It should be noted that the results obtained must be viewed critically in terms of their transferability to a larger population. One limitation, for example, is that only a limited number of children and young people are active singers, who may differ in their singing techniques. In addition, the selection of the adolescents for this study is not representative of children and young singers in general in terms of the development of vocal skills and cognitive abilities during growing up.

**Fig 5:**
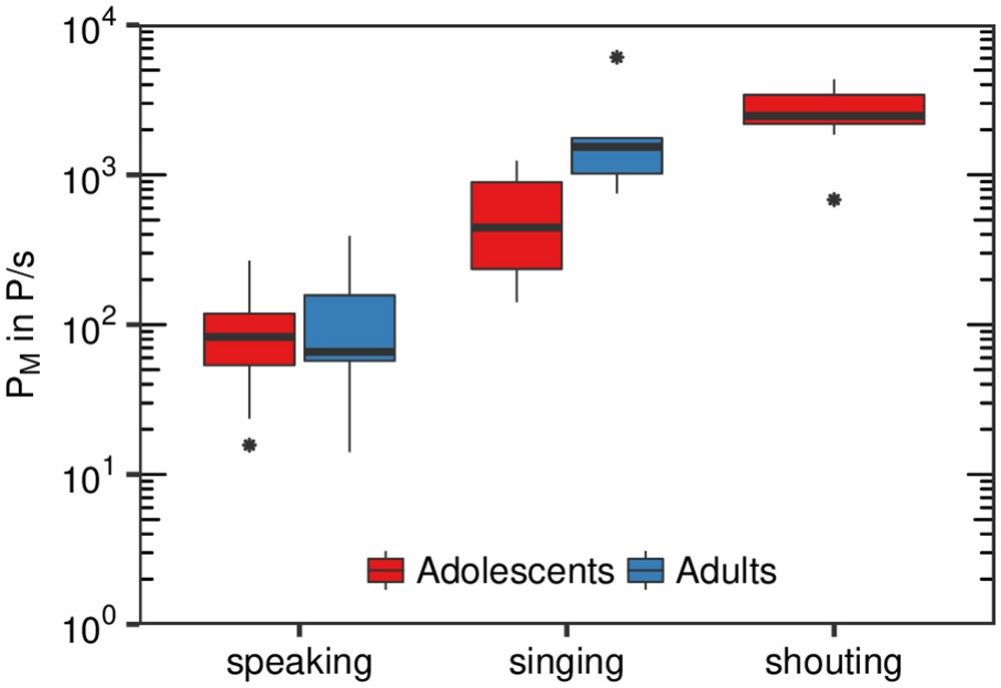
Comparison with data of adults singers. Boxplots of subject specific median emission rates (P_M_ in P/s) for the test conditions speaking, singing and shouting (adolescents only). Present data of young voices (n = 8) in comparison with previously measured corresponding data of professional adult singers (n = 8) (http://doi.org/10.5281/zenodo.4011701, see [4]).

Comparing these values with previously published data for adult professional singers [4](http://doi.org/10.5281/zenodo.4011701) using the same experimental setup, similar values for speaking, but lower values for singing were observed (Fig 5). For singing, the ratio in medians between adults and adolescents was about 3.1. Shouting values for adolescents (not available for adults) were higher than singing values for adults. Regarding sustained phonation with loud voice, the ratio in medians between adults and adolescents for the forte condition was about 6.8. It must be noted that there were slight deviations between adults and adolescents in the execution of this task, such as adolescents were allowed to shortly breathe within the recording sequence. Except for shouting, determined values for adolescents are also lower than recently published data found in professional and non-professional adult singers [3]. In this study of 12 adult subjects, emission rates of 320-2870 P/s for singing at moderate to loud volumes are determined. On the other hand, Morawska et al. [9] reported values for 15 adults of 0.322 to 1.088 P/cm^3^ for voiced speech and normal speaking that can be approximately converted to emission rates by multiplying these values with a mean inhalation rate for males and females of about 9.5 l/min (see [10], p.18), to about 51 to 172 P/s. In a comprehensive study with 48 adult subjects, Asadi et al. [5] found loudness correlated emission rates of 1 to 50 P/s for normal speech. Further, Gregson et al. [11] estimated also loudness dependent particle concentrations in the order of 0.1 to 1.3 and 0.19 to 2.47 P/cm^3^ (corresponds to an emission rate of about 15.8 to 391.1 P/s) in a study with 25 adult subjects for speaking and singing tasks. Quite lower values for adults in the order of 0.0049 to 0.0215 P/cm^3^ (corresponds to an emission rate of about 0.8 to 3.4 P/s) for talking tasks were also reported [12]. It can be summarized, that the adolescent’s data for the speaking tasks presented here, are in the same order of magnitude than values reported earlier.

Regarding particle size distributions, it was found in several studies, that these distributions are mostly centered in a region of about 0.5 to 2 μm for singing [3, 11], and 0.8 to 1 μm for speaking [3, 5] and further skewed to smaller particle size. These observations were confirmed by our data for adolescents (see Fig 2).

There might be different reasons for the lower emission rates for child voices during singing. Before puberty voice changes, there are considerable differences in the vibration characteristics of the vocal folds in comparison to adults. Typical features of a child’s vocal register in singing include differences in contact time and contact area of the vocal folds during each vibration cycle. There are also differences in the subglottic pressure between adults in general and young people [13, 14]. Further, there are smaller anatomical proportions of the child’s airways and vocal folds are shorter before puberty voice changes. On the other hand, the fundamental frequency of the voice and accordingly the contact frequency of the vocal folds might be higher, especially in comparison with male adult voices.

Indeed, a major reason for the lower emission rates in comparison to professional singers might be the lower volume of the adolescent’s voices during singing. This was especially evident in the task with intended loud singing, even if all subjects of this study had a longstanding choir experience. On the contrary, in the shouting condition, which is not related to limitations in the child’s singing technique, some adolescents reached higher emission rates than adults during loud singing.

Our experimental setup uses a Laser Particle Counter (LPC) in cleanroom conditions (without a background concentration of particles) to assess the number and size of evaporated aerosols (or droplet nuclei) in their equilibrium state for different kinds of vocalisation. Estimating the precise number and size of these particles is of great interest to assess the concentration of those particles in closed rooms. Because of the great range of the exhaled air volume flow from zero (close mouth) up to at least 7.5-15 l/min for phonation [10, 15–17] and 24 l/min for blowing [18], the measurement setup must be both, highly sensitive to detect all particles and suitable to cover the whole volume flow range.

Thus, different considerations were included in designing the setup for this study. First of all, a filter fan unit with a high volume flow of 400 m^3^/h has been selected, whereas the flow of exhaled air is small and can be neglected. To further avoid any disturbances regarding stagnating flow at the measuring probe, this probe was positioned centrally in a glass pipe. Further, by placing a turbulence generating baffle between mouth opening and probe, there was a homogenous particle density distribution in the cross sectional area of the glass pipe. This in turn required the choice of an adequate distance between mouth opening and LPC, which was chosen to about 0.81 m and resulted in a traveling time of the particles of about 0.14 s in maximum (see [4] for details). These experimental conditions, including a relative humidity of about 46%, result in approximately evaporated aerosols in their equilibrium state [19], which can be surveyed by the LPC with high accuracy and independently of the fluid flow at the mouth. Thus, the measured emission rates can serve as a realistic estimate for a possible carriage for viruses that propagate in the environment. Moreover, they allow a reliable comparison between the different vocalisation tasks. However, the emission rates reported in this study should not be interpreted as emitted droplets and aerosols directly at one’s opened mouth [20]. Further issues with relevance for SARS-CoV-2–transmission during singing like the trajectory of larger droplets after emission from the mouth need to be studied with different methods like Particle Image Velocimetry or Phase Doppler Anemometry.

For the assessment of the risk of SARS-CoV-2-transmission during singing, both, droplets and aerosols are considered as virus carriers. While virus transmission via droplets can be mainly handled by distance and hygiene rules, the risk management of transmission through virus carrying aerosols has to be addressed with further strategies [21–23].

Activities to reduce the aerosol input in closed rooms during singing include limiting the number of singers and the rehearsal or performance time, which contributes to a lower cumulative aerosol concentration. Apart from these issues, the individual emission rates of the singers determine the aerosol input into closed rooms. For singing, an increased rate of aerosol emission compared to speaking has been found for adolescents, too. The lower aerosol emissions for adolescents’ voices during singing in comparison to adult singers might contribute to develop more specific risk management strategies for different constellations of singing. Apart from singing, adolescents’ aerosol emissions during shouting may be even higher than adult’s emission rates during speaking. That should be considered for risk assessments in the corresponding areas, too.

Further, risk management strategies should incorporate other approaches like room size and air condition systems, which will affect the number and the concentration of potentially infectious aerosols in the room, too. Especially modern mechanical ventilation systems might significantly lower the risk of aerogenic virus transmission [24].

Based on the current prevalence of the disease, advanced risk management for singing together for instance in music lessons in school should combine the above-mentioned tools. The findings for aerosol emission for adolescent voices should be especially used to specify rehearsal and performance schedules for children’s and adolescents’ choirs because of the significance of education and socio-emotional development for children and young people.

## Supporting information

S1 File Raw data. R-readable data frame. In addition to data for ID, condition, cumulative PM, and SPL, the emission rates for the six size classes (C1-C6), corresponding to >0.3–0.5, >0.5–1.0, >1.0–3.0, >3.0–5.0, >5.0–10.0, and >10.0–25 μm), are given.
(CSV)

S2 File Statistical analysis. R-script for running the statistical analysises of the data provided in S1 File.
(RMD)
